# Specific Remodeling of Splenic Architecture by Cytomegalovirus

**DOI:** 10.1371/journal.ppat.0020016

**Published:** 2006-03-03

**Authors:** Chris A Benedict, Carl De Trez, Kirsten Schneider, Sukwon Ha, Ginelle Patterson, Carl F Ware

**Affiliations:** Division of Molecular Immunology, La Jolla Institute for Allergy and Immunology, San Diego, California, United States of America; Robarts Research Institute, Canada

## Abstract

Efficient immune defenses are facilitated by the organized microarchitecture of lymphoid organs, and this organization is regulated by the compartmentalized expression of lymphoid tissue chemokines. Mouse cytomegalovirus (MCMV) infection induces significant remodeling of splenic microarchitecture, including loss of marginal zone macrophage populations and dissolution of T and B cell compartmentalization. MCMV preferentially infected the splenic stroma, targeting endothelial cells (EC) as revealed using MCMV-expressing green fluorescent protein. MCMV infection caused a specific, but transient transcriptional suppression of secondary lymphoid chemokine (CCL21). The loss of CCL21 was associated with the failure of T lymphocytes to locate within the T cell zone, although trafficking to the spleen was unaltered. Expression of CCL21 in lymphotoxin (LT)-α–deficient mice is dramatically reduced, however MCMV infection further reduced CCL21 levels, suggesting that viral modulation of CCL21 was independent of LTα signaling. Activation of LTβ-receptor signaling with an agonistic antibody partially restored CCL21 mRNA expression and redirected transferred T cells to the splenic T cell zone in MCMV-infected mice. These results indicate that virus-induced alterations in lymphoid tissues can occur through an LT-independent modulation of chemokine transcription, and targeting of the LT cytokine system can counteract lymphoid tissue remodeling by MCMV.

## Synopsis

The architecture of the spleen and lymph nodes is organized into discrete compartments that promote the immune system's ability to capture pathogens and mount protective defenses. The white blood cells that fill these compartments are positioned in discrete regions by chemoattractants produced by the cells that form the architecture of the spleen (stroma). The white blood cells must be positioned to interact with each other to mount effective host defense. Benedict and colleagues demonstrate that cytomegalovirus, a β herpesvirus, can remodel the architecture of the spleen by specifically inhibiting a key chemoattractant produced by the stromal cells, the chemokine CCL21. This action by the virus impedes efficient contact between cells by altering the movement of T lymphocytes to their correct position within the spleen. However, as a counter strategy, activating the lymphotoxin-β receptor pathway, a cytokine known to control the embryonic development of lymphoid organs, partially restored CCL21 and the ability of T lymphocytes to find their correct position. Thus, infection with this herpesvirus selectively remodels the structure of lymphoid tissues perhaps aiding in the formation of a portal though host defenses.

## Introduction

The microarchitecture of secondary lymphoid organs, like the spleen, facilitates effective communication between antigen-presenting cells and T lymphocytes to mount protective immunity to pathogens. The white pulp of the spleen is organized around a central arteriole and is demarcated from the surrounding red pulp by the marginal sinus defining the marginal zone (MZ), wherein antigen-capturing macrophages, B cells, and dendritic cells (DC) reside [1]. Two distinct macrophage populations that are present in the MZ include marginal zone macrophages (MZM) and marginal metallophilic macrophages (MMM), and these cells localize to the outer and inner border of the MZ, respectively. Within the white pulp, T and B lymphocytes are also segregated into discrete compartments, with T cells comprising the periarteriolar lymphoid sheath (PALS) surrounded by the B cell follicles.

The localization and subsequent organization of T and B lymphocytes to the white pulp is regulated through their interactions with unique populations of chemokine-producing stromal cells [2]. Naive T lymphocytes express high levels of the chemokine receptor CCR7, and upon entering the spleen they migrate toward, and congregate upon, stromal cells that produce the secondary lymphoid tissue chemokines CCL21 (SLC/6Ckine/Exodus-2/TCA4;) and CCL19 (ELC/MIP-3β) [3,4]. Two distinct isoforms of CCL21, encoded by separate gene products that differ by the presence of a serine or leucine at amino acid residue 65, are expressed in C57BL/6 mice. CCL21-ser(a) appears to control the migration/localization of T cells and DC in lymphoid organs [5,6]. Importantly, CCL21-ser regulation of cellular trafficking seems to be due to its high expression levels compared with CCL21-leu(b) in lymphoid tissues, and not a functional difference between these two isoforms in mediating cellular chemotaxis [7]. In contrast, all mature B cells express the chemokine receptor CXCR5, and migrate in response to the production of B lymphoid chemoattractant CXCL13 by radio-resistant stromal cells present in the B cell follicle [8,9].

The development and maintenance of chemokine-expressing stromal cells requires signaling by members of the TNF family of cytokines, lymphotoxin (LT)α (TNFSF1), LTβ (TNFSF3), and TNF (TNFSF2) [10], most likely through their activation of transcription factors in the NFκB family [11]. The role of LTαβ-LTβ receptor (LTβR, TNFRSF3) signaling in regulating lymphoid tissue chemokine expression at the developmental, neonatal and adult stage in mice is complex and is still being dissected [2]. Nevertheless, triggering LTβR signaling can induce expression of CCL21 in the spleen of adult mice via activation of the “noncanonical,” IKKα/NIK-dependent NFκB pathway [12]. Interestingly, LTαβ signaling has been reported to be critical for the specific induction of CCL21-ser in a mouse model of lung inflammation [13]. However, CCL21-ser induction and the subsequent formation of tertiary lymphoid structures occurs in the lungs of LTα–deficient mice infected with influenza, indicating LTαβ regulation of CCL21-ser is not absolute [14].

Herpesviruses, such as cytomegalovirus (CMV), are effectively controlled in the immunocompetent host, with infection resulting in little overt pathogenicity. However, despite effective host immune defenses, herpesviruses establish a lifelong, persistent infection. Studies of mouse CMV have provided an insightful model, revealing the importance of innate defenses in the control of acute infection, particularly type 1 interferons and specific NK cell subsets [15–17], and adaptive immunity mediated by T lymphocytes required for viral clearance and control of latency [18]. Recently, we uncovered a role for the LTαβ cytokine system in regulating the early type 1 IFN response during MCMV infection, and identified this “cytokine axis” as essential for survival of T and B cells [19,20].

These results suggested that CMV might alter the pathways controlling the organization of lymphoid tissue as a strategy to impede efficient trafficking and intercellular communication between responding host cells. Here we show that MCMV disrupts the compartmentalization of lymphocytes and alters trafficking of adoptively transferred T cells by inducing the specific downregulation of CCL21 mRNA in splenic stromal cells. T cell trafficking to the splenic T cell zone is partially restored by activating signaling via the LTβR, indicating that MCMV targets chemokine-controlled architecture as part of its manipulation of host defenses.

## Results

**Figure 1:**
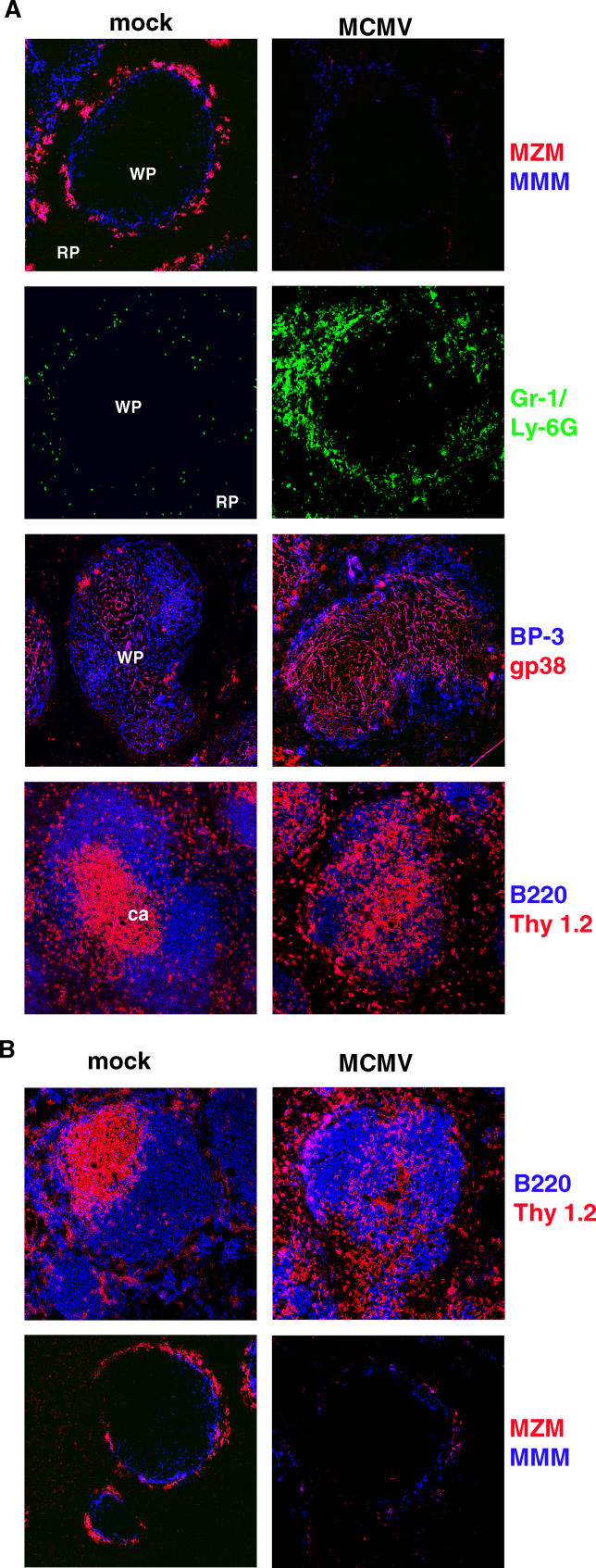
MCMV Modulates Splenic Architecture (A) Spleens from PBS (mock) or MCMV-infected B6 mice (3.2 × 10^5^ pfu) were harvested at day 3 post-infection for immunohistochemical analysis. Spleen sections were incubated with markers specific to MMM (MOMA-1, MMM, blue), MZM (ER-TR9, MZM, red), granulocytes (Gr-1/Ly-6G, green), stromal cell populations (BP-3, blue, to identify B-cell-zone stromal cells and gp38, red, to identify T-cell-zone stromal cells, in uninfected mice), and B (B220, blue), and T lymphocytes (Thy1.2, red) (magnification 20×). These sections are representative of four independent experiments with *n* = 3 mice per group with two or more sections taken from each spleen. White pulp (WP) and red pulp (RP) and the location of the central arteriole (ca) are indicated. (B) Similar immunohistochemical analysis performed on Balb/c mice, 3 d post-MCMV-infection (1 × 10^5^ pfu).

To examine whether MCMV infection might modulate lymphoid-tissue organization, splenic architecture was examined by immunohistochemistry during acute viral infection. Infection of C57BL/6 (B6) mice with MCMV induced striking changes in the MZ and white pulp areas by 3 d post-infection (Figure 1A). MCMV caused a complete loss of MZM and a reduction in the MMM, identified by the ER-TR9 and MOMA-1 markers, respectively. Indicative of virus-induced inflammation, granulocytes (Gr-1/Ly6G^hi^ and CD11b^hi^) accumulated in the splenic MZ and red pulp within 48–72 h. Evidence that stromal cell populations in the spleen were altered during MCMV infection was also seen. T cell zone stromal cells can be identified by the expression of gp38 (T1α/podoplanin) [21], a cell surface mucin required for the appropriate development of the lymphatic endothelium [22]. After MCMV infection, an abnormally broad pattern of gp38 staining was observed throughout the white pulp, which in uninfected mice is restricted to the PALS (Figure 1A). By contrast, the BP-3 (BST-1/CD157; NP_033893) marker identifying stromal cells located mainly in the B cell follicle remained unchanged. Perhaps most conspicuously, B and T lymphocytes were no longer compartmentalized into distinct regions in the white pulp. The effects on microarchitecture progressed in severity as the virus inoculum was increased (unpublished data), with changes evident by 48 h and maximal at 72–96 h, concurrent with the peak of MCMV replication in the spleen of B6 mice [23]. Moreover, these changes did not occur when inactivated (UV-irradiated) virus was used to infect the mice (unpublished data), indicating that either viral gene expression or productive replication is required for modulation of splenic architecture. B6 mice are relatively resistant to MCMV infection, primarily through the action of the *cmv1* dominant-acting resistance gene encoding the Ly49H-activating receptor expressed on a subset of NK cells, which help to control viral replication in the spleen [17,24,25]. Importantly, similar modulation of splenic architecture was also seen in Balb/c mice infected with MCMV, indicating that Ly49H+ NK cells were not required for the observed alterations (Figure 1B).

**Figure 2:**
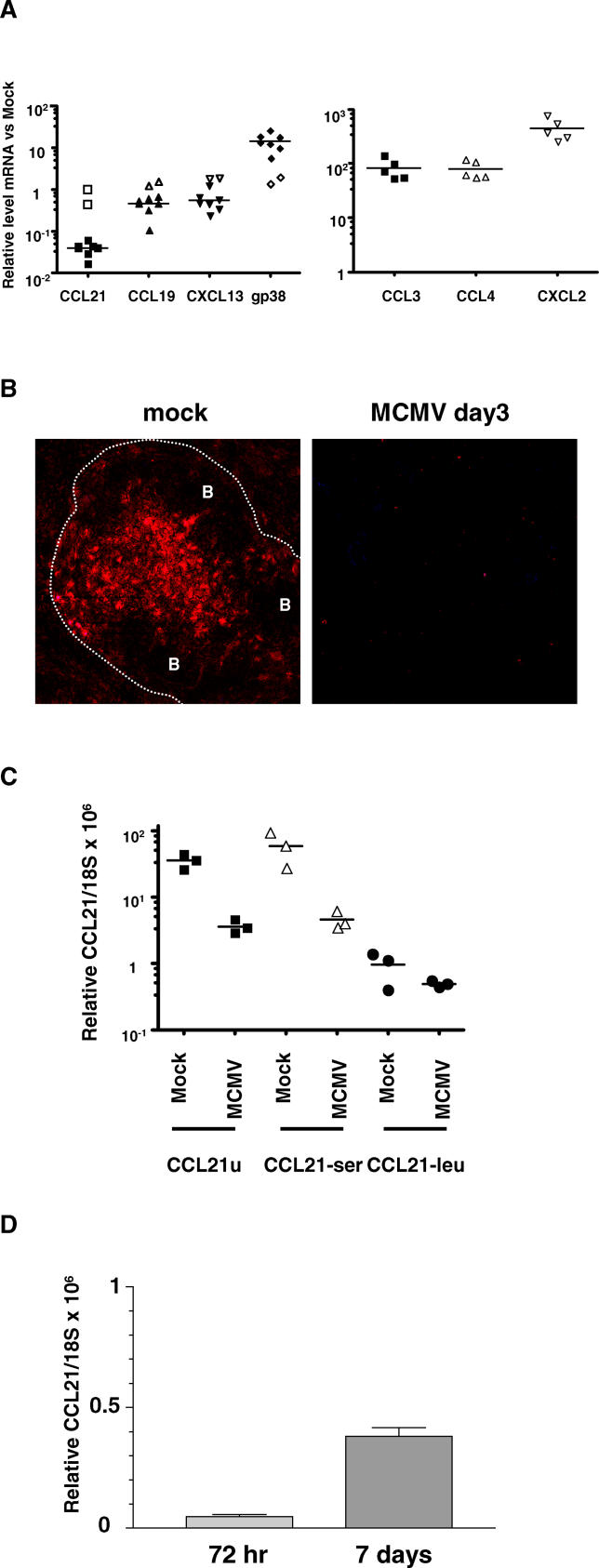
Reduced CCL21-ser Expression after MCMV Infection Spleens from B6 mice mock-infected or infected with 3.2 × 10^5^ (A,B,D) or 1.5 × 10^5^ (C) pfu of MCMV were harvested at day 3 post-infection. Gene expression was measured by quantitative PCR and was normalized to 18S RNA and presented as relative mRNA levels compared to uninfected mice (A and D) or as absolute values (C). When presented as relative values, mRNA levels from three uninfected mice (mock) were determined and averaged for comparison for each experiment. Open symbols in (A) represent mice infected with UV-inactivated MCMV (equivalent to 3.2 × 10^5^ pfu). (B) Immunohistochemical analysis of CCL21 expression in the spleen of a mock or MCMV-infected B6 mouse 3 d post-infection; the location of the periarteriolar lymphoid sheath “PALS” and B-cell follicles “B” is noted, and the interrupted white line indicates the outer edge of the MZ. This section is representative of three experiments (*n* = 3 mice per group). In (C), primers used for analysis of CCL21 levels amplified either the serine (CCL21-ser), leucine (CCL21-leu), or both (CCL21u) isoforms. The data in (D) is an average of three infected mice ± SEM (*p* < .001).

The specific changes observed in B and T lymphocyte segregation and the alteration of stromal cells populations in the spleen suggested that expression of the lymphoid tissue organizing chemokines might be altered upon infection with MCMV. Indeed, CCL21 mRNA was specifically decreased in the spleens from B6 mice infected with MCMV (Figure 2A) with a corresponding loss of CCL21 protein staining in the T cell zone (Figure 2A and 2B). Although stromal cells in the T cell zone coexpress CCL19 and CCL21, the levels of CCL19, and CXCL13 (expressed by stromal cells in the B-cell zone), were largely unaffected after MCMV infection (Figure 2A). In contrast, expression of several inflammatory chemokines (CCL3, CCL4, and CXCL2) were all highly elevated (Figure 2A), consistent with the observed recruitment of granulocytes into the spleen [26]. Interestingly, the decreased CCL21expression after MCMV infection was due to a specific effect on the serine isoform of CCL21 (CCL21-ser) (Figure 2C). The decrease in CCL21-ser was also viral replication dependent, as infection with UV-inactivated MCMV did not result in modulation of CCL21 mRNA levels (Figure 2A). However, CCL21 mRNA recovered to levels near that of uninfected mice by day 7 post-infection, a time point when virus production has been largely controlled in the spleen (Figure 2D). These results indicate that MCMV infection results in a specific, but transient, inhibition of the CCL21 chemokine system.

In the spleen, stromal cells in the T-cell zone appear to be the primary producers of CCL21 [2,9]. Stromal cells in the T cell zone of uninfected mice coexpress these two proteins [27]. Interestingly, gp38 mRNA was strongly upregulated following MCMV infection (8-fold to 20-fold) (Figure 2A). These results suggest that MCMV infection does not compromise the viability of CCL21/gp38 coexpressing stromal cells, but instead selectively alters CCL21-ser transcription. Alternatively, since gp38 expression was no longer restricted to the PALS, and an intact T cell zone was also absent, it is possible that the increase in gp38 expression occurred on other cell types after MCMV infection.

MCMV dependent modulation of gp38 and CCL21-ser transcription in total spleen RNA indicated that infection could alter gene expression in the stroma. To identify virus-infected cells, a recombinant MCMV that expressed green fluorescent protein (MCMV-GFP) [28] was utilized. This recombinant virus showed an equivalent capacity to induce loss of CCL21 as the Smith strain of MCMV (unpublished data). Flow cytometry revealed that the distribution of cells displaying GFP fluorescence in the hematopoietic compartment at 48 h and 72h post-infection were granulocytes (~18% and 14% at 48 h and 72 h, respectively), macrophages (~10% and 6%), NK cells (~3% and 11%), and DC (~6% and 3%), but very few T or B lymphocytes (< 2% of each) (Table 1). To accurately determine these percentages, it was necessary to compare the fluorescence of individual hematopoietic cell subsets in the FL-1 channel from mice infected with MCMV-GFP to cells from mice infected with MCMV-Smith due to changes in the autofluorescence of different cellular subsets following virus infection (see Figure S1, specifically Figure S1C). In addition, the expression of the macrophage marker F4/80 was downregulated in mice infected with MCMV, as has been reported previously [29], and this was accounted for when assessing the percentage of infected macrophages (see Figure S1A and S1B).

**Table 1:**
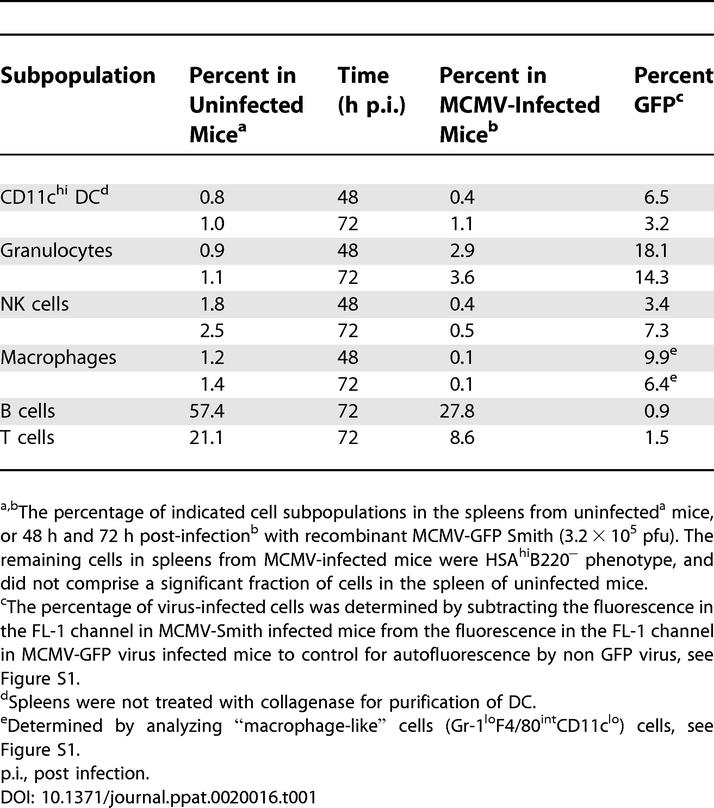
Splenic Subpopulations after Infection with MCMV

**Figure 3:**
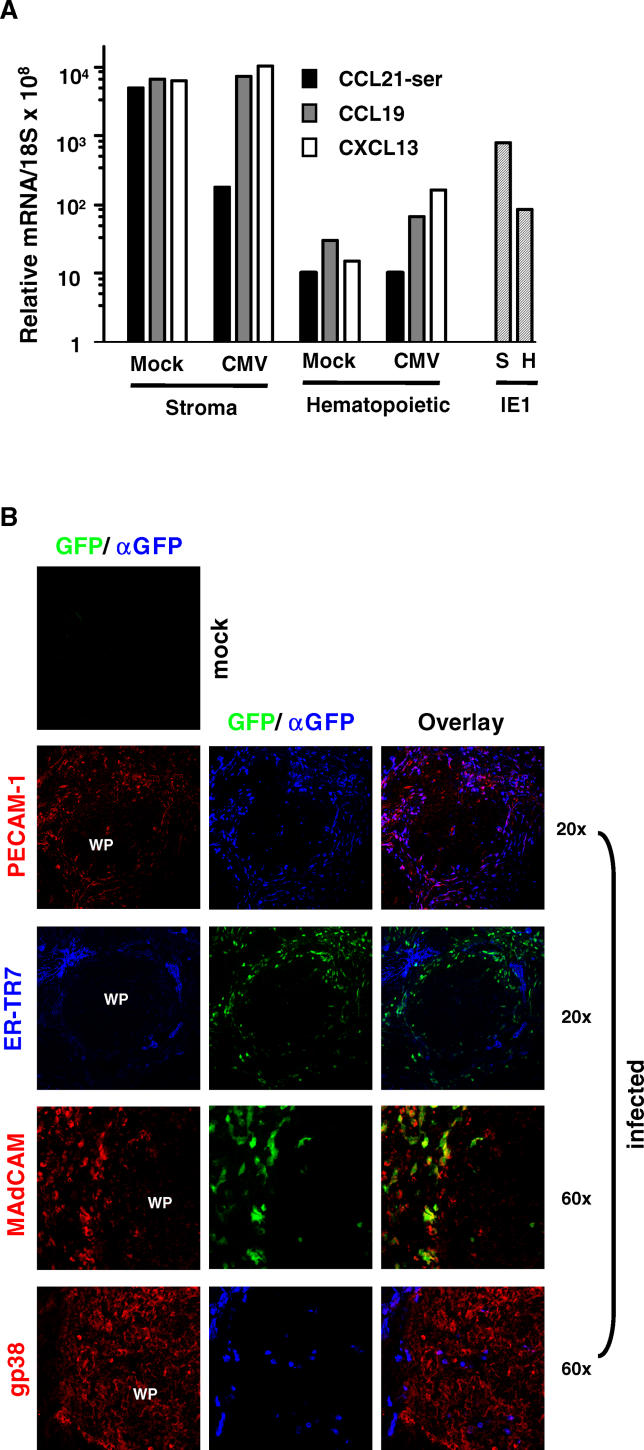
MCMV Splenic Tropism All analysis was performed 3 d post virus infection. (A) Splenic stromal cells from mock or MCMV (2 × 10^5^ pfu, Smith strain) infected spleens were separated from hematopoietic cells by extrusion through a nylon mesh filter (see Materials and Methods). Total cell RNA was then isolated from either the stromal cells (S) or hematopoietic cells (H) fractions, and quantitative RT-PCR was performed to detect expression of CCL21-ser, CCL19, and CXCL13 as well as expression of the MCMV immediate early-1 (IE1) transcript. (B) Spleens from MCMV-GFP infected mice (2 × 10^5^ pfu) were examined by immunohistochemistry. Panels are color-coded with the text for the antigen examined, and field magnification is shown to the right of the panels. No background staining was seen in uninfected mouse spleen when directly detecting GFP expression or when using an anti-GFP antibody (αGFP) for signal amplification. WP, white pulp.

To quantify the relative tropism of MCMV for the splenic hematopoietic and stromal cells, the spleen was separated into two fractions by extrusion through a nylon filter. An ~500-fold enrichment of mRNA for CCL21 and CXCL13, and ~200-fold for CCL19 was observed in the fraction retained by the filter (stroma) when compared to hematopoietic cells in the extruded fraction from uninfected mice (Figure 3A). MCMV infection altered CCL21-ser expression (28-fold decrease) in the stromal fraction, with no reduction in CCL19 or CXCL13 expression. Viral gene expression as determined by analysis of IE1 (immediate early gene-1) largely fractionated with stromal cells (~9-fold abundance over the level of IE1 in the hematopoietic compartment) (Figure 3 A). Together, these data indicate that MCMV preferentially infects the splenic stromal compartment, and suppressed CCL21-ser. To further map the location of MCMV-infected cells in the spleen, a detailed immunohistochemical analysis was performed (Figure 3B). The majority of cells expressing high levels of GFP were localized in the red pulp and MZ region of the spleen. Many GFP^+^ cells in the red pulp costained for platelet–endothelial-cell adhesion molecule-1 (PECAM-1), but not for a marker of the reticular fibroblastic network (ER-TR7), suggesting that the virus infected EC. High magnification analysis in the MZ revealed that some GFP-expressing cells also co-expressed mucosal addressin cell adhesion molecule (MAdCAM), while other cells displayed morphology typical of GR-1+ granulocytes previously localized to this region (see Figure 1). Low-magnification analysis revealed a sparse distribution of GFP-expressing cells in the splenic white pulp compared with the MZ and red pulp. When the white pulp area was examined at higher magnification, the GFP^+^–MCMV-infected cells were morphologically hematopoietic cells without typical stromal cell characteristics. Although gp38 levels were upregulated in the white pulp of virus-infected mice (see Figure 1), a small fraction of cells coexpressing gp38 and GFP were sporadically observed (Figure 3B). These data indicate that MCMV preferentially infects splenic endothelium and hematopoietic cells localized in the red pulp and MZ.

**Figure 4:**
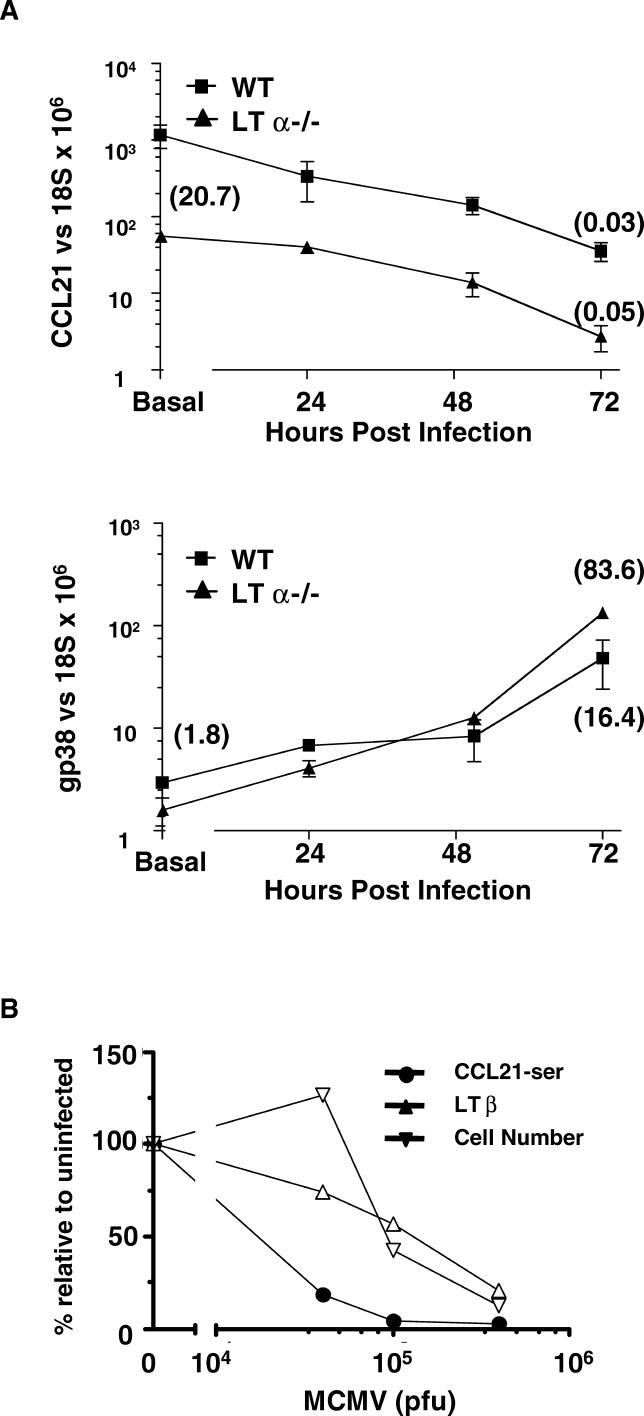
MCMV-Induced Suppression of CCL21 Is Independent of LTαβ-LTβR Signaling LTα-deficient or wild-type (B6) mice were infected with MCMV (Smith strain, 3.2 × 10^5^ pfu), and spleens were harvested at various times post-infection for analysis of CCL21 and gp38 levels by quantitative PCR. Gene expression levels displayed on the *y*-axis are the levels in uninfected mice, and fold reduction in gene expression in uninfected LTα^−/−^ compared with wild-type is shown in the parentheses near the *y*-axis. Values in parentheses at the right of the figure represent the fold reduction in gene expression 3 d post-virus-infection compared with uninfected mice of the same genotype. (B) B6 mice were infected with various amounts of MCMV (2 × 10^4^, 1 × 10^5^, 3.2 × 10^5^ pfu), and spleens were harvested 3 d post-infection for analysis of CCL21-ser and LTβ mRNA levels by quantitative PCR. Mice were infected in parallel with the same doses of MCMV for analysis of total splenocyte numbers 3 d post-infection.

LTαβ–LTβR signaling is required for the development of CCL21 and gp38-expressing stromal cells in the spleen [30], which prompted us to examine the effect of MCMV infection on CCL21 and gp38 expression in the spleens of LTα-deficient mice (Figure 4A). Although LTα-deficient mice have significantly lower basal levels of CCL21 expression when compared with wild-type mice (~20-fold reduced), MCMV infection resulted in a further decrease (20-fold), occurring over a similar timecourse to that seen in control mice. Similar to wild-type mice, the CCL21ser isoform was specifically affected by MCMV infection (unpublished data). By contrast, the basal transcription level of gp38 was modestly reduced in spleens of uninfected LTα-deficient mice compared with wild-type mice (1.8-fold reduced compared with wild-type), yet 3 d after MCMV infection gp38 was dramatically upregulated in both (16-fold in wild-type; 84-fold in LTα−/−). Additionally, the expression of LTβ in total spleen mRNA was determined 3 d post-infection in wild-type mice infected with various amounts of MCMV (Figure 4B). At lower doses of virus (2 × 10^4^ pfu), CCL21-ser expression was significantly reduced (~5-fold), but expression of LTβ was minimally affected (~20% reduction). With increasing inoculum of MCMV, CCL21-ser levels further decreased, as did LTβ expression. However, at the two higher doses of MCMV the reduction in LTβ was proportional to the loss of total splenocytes (hematopoietic cells) (Figure 4B), suggesting that the reduction in LTβ mRNA probably reflected a loss of cells. This loss of lymphocytes also contributes to the alteration in the relative percentages of hematopoietic cell subsets observed in the spleen after MCMV infection (see Table 1). This lymphocyte “death” may be linked to LTαβ-LTβR signaling required for sufficient induction of IFNβ, which contributes to lymphocyte survival [20]. These results suggest that the MCMV-induced effects on CCL21 and gp38 expression can occur largely in the absence of an intact LTαβ cytokine system, and that reduced LTαβ expression is likely not responsible for the decrease in CCL21-ser.

**Figure 5:**
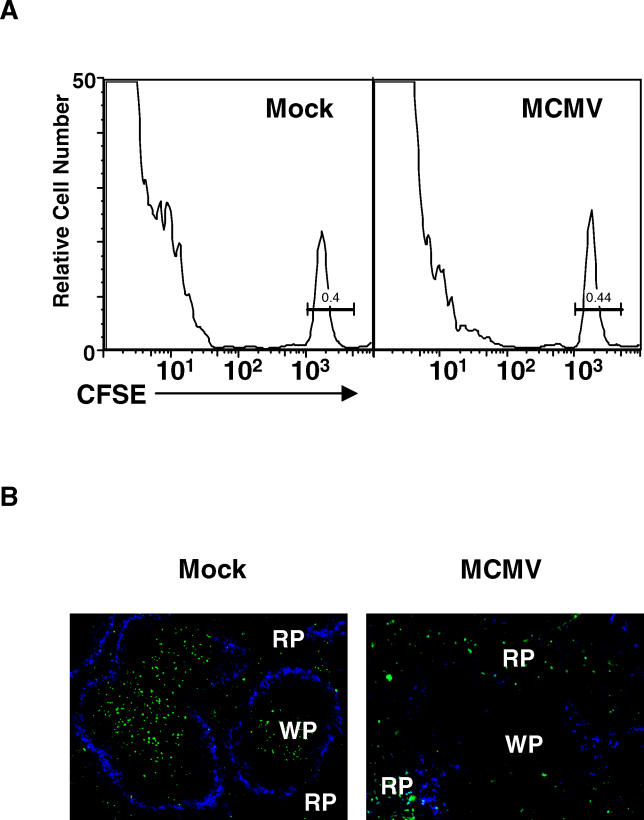
MCMV-Induced Suppression of CCL21 Abolishes T Cell Localization to the White Pulp Mock- or MCMV-infected B6 mice (1.5 × 10^5^ pfu) were injected with CFSE labeled naive T cells, and spleens were removed for analysis 2 h later. (A) Flow cytometry was used to assess the total number of CFSE-labeled cells in the spleen after adoptive transfer into mice 3 d post-infection. (B) Immunohistochemical analysis of T cell localization in the spleen at 3 d post-infection using the MOMA-1 marker to identify MMM (shown in blue, magnification 10×). WP, white pulp; RP, red pulp.

It is not clear whether the main effector chemokine that controls T cell localization to the PALS is CCL19 or CCL21. The specific suppression of CCL21-ser expression, but not CCL19 expression, after MCMV infection, presented an opportunity to distinguish the function of these two chemokines. MCMV infection should impair the homing of T cells to the PALS if CCL21 is the critical chemokine regulating this trafficking. To assess T cell trafficking in vivo, flow cytometry and immunohistochemistry were used to monitor the movement of adoptively transferred T cells labeled with a fluorescent dye (CFSE) (Figure 5). Naive T cells transferred into mock-infected mice showed rapid localization (within 2 h) to the PALS, while T lymphocytes transferred into MCMV-infected mice were unable to home to the T cell zone (Figure 5B), although equivalent numbers of labeled T cells from either mock- or MCMV-infected mice migrated to the spleen (Figure 5A). Importantly, T cells isolated from MCMV-infected mice were fully capable of localizing to the PALS when injected into uninfected mice, indicating that the function of the CCL21 receptor (CCR7) was unaffected in splenic T cells after MCMV infection (unpublished data). However, T cells transferred to MCMV-infected mice were largely restricted to the MZ and red pulp (Figure 5B), similar to the trafficking patterns of T cells in paucity-of-lymph-node–T cells *(plt)* mice (which lack expression of CCL21-ser and CCL19) and CCR7-deficient mice [3,4].

**Figure 6:**
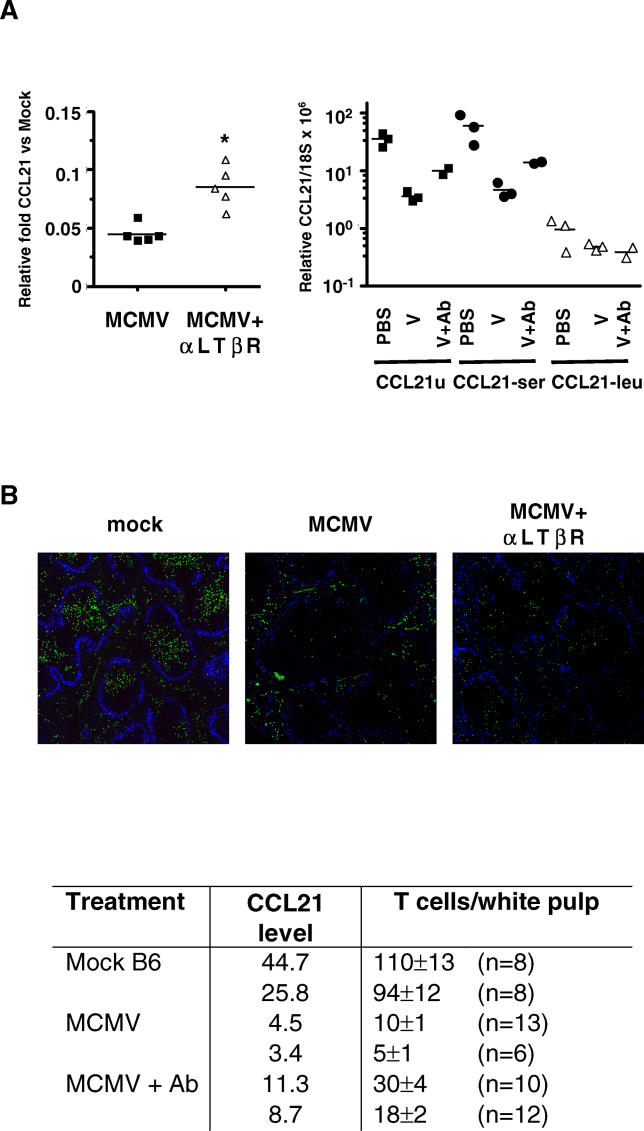
Pharmacologic Activation of LTβR Signaling Partially Restores CCL21 Expression and T Cell Localization in the Spleen of MCMV-Infected Mice (A) Quantitative PCR was used to determine the relative levels of CCL21 gene expression 3 d post-MCMV infection in spleens from control- or virus- (V) infected mice ± agonistic anti-LTβR antibody (Ab) treatment. Left panel, 3.2 × 10^5^ pfu of MCMV was used for infection, and only total CCL21 mRNA levels were analyzed using CCL21u primers (see below). Right Panel, mice were infected with 1.5 × 10^5^ pfu MCMV and expression of the various CCL21 isoforms was determined. CCL21u, universal CCL21 primers that amplify both the leucine (leu) and serine (ser) isoforms; *p* = 0.02 (B) Shown are representative spleen sections from the same mice that were analyzed in the right panel of (A). Sections were incubated with an antibody to detect MMM (MOMA-1, MMM, blue), and adoptively transferred, CFSE-labeled T cells are green. The number of CFSE labeled T cells located per WP area after transfer was analyzed (*n* = number of WP regions analyzed per spleen), and differences between groups of infected mice ± antibody is statistically significant to a *p-*value < 0.0001.

The observation that MCMV modulation of CCL21 and gp38 transcription occurred in both wild-type and LTα-deficient mice suggested that the LTαβ-CCL21 signaling “axis” might still be intact in the lymphoid tissue of these infected mice. To test whether this pathway was active, we used an agonistic antibody that activates LTβR signaling [12,20] to induce CCL21 expression. The anti-LTβR antibody was co-injected at the time of MCMV infection. CFSE-labeled T cells were adoptively transferred 3 d post-infection, and spleens were harvested for analysis of T cell localization and CCL21 expression. A single injection of anti-LTβR resulted in a modest restoration of CCL21 mRNA levels (~2-fold), without any change in CCL19 or CXCL13, which was restricted to the CCL21-ser isoform (Figure 6A). A commensurate increase in the localization of transferred T cells to the white pulp also occurred in anti-LTβR–treated mice (Figure 6B). This result indicates that the LTβR pathway remains operable during MCMV infection. Activating the LTβR pathway was sufficient to direct trafficking of T cells to the PALS counteracting viral-induced alterations in lymphoid tissues. The induction of CCL21 by the LTβR agonist suggests that the MCMV-induced decrease in CCL21-ser expression is responsible for the inability of transferred T cells to localize to the PALS.

## Discussion

The cytokine pathways required for the development and maintenance of organized lymphoid tissue also contribute to efficient host defense against viral pathogens. Our previous observations that MCMV exhibited increased virulence in mice lacking an intact LTαβ-cytokine system [19,20], a system critical for the development and maintenance of lymphoid tissue architecture, prompted us to examine whether viral infection might alter the structure of lymphoid organs in wild type mice. MCMV infection induced several prominent changes in the microarchitecture of the spleen, including alterations in MZ macrophage populations, a complete loss of B and T cell segregation in the white pulp commensurate with a specific and dramatic loss in CCL21-ser expression, and increased expression of the PALS stromal cell marker gp38. We identified the PECAM-1–and MAdCAM-expressing EC as major cellular targets of the splenic stroma infected with MCMV. The EC expressing these markers in the MZ contribute to the “threshold” that T cells must cross to accumulate in the PALS after entry into the spleen through the MZ sinus. Regulation of T cells trafficking is accomplished in part through the expression of key integrins and chemokines, including CCL21 [31].

The phenotype of mice deficient in CCR7 [3] or harboring the mutation *plt,* which lack CCL21-ser and CCL19 [6,27], provided solid evidence that these chemokines regulate T cell trafficking to the white pulp and maintenance of T and B cell segregation. Additionally, T/B cell zones are disorganized in the spleens of mice deficient in LTαβ signaling, which also show marked reductions in CCL19 and CCL21-ser expression and CXCL13 [13,32]. Unfortunately, the construction of mice singly deficient in either CCL21-ser or CCL19 signaling has not been achieved. Here we identified that the β-herpesvirus CMV can in a highly specific fashion inactivate CCL21-ser at the transcriptional level. MCMV had no effect on CCL21-leu or CCL19. Infection with MCMV induced a blockade in T cell trafficking to the PALS, implicating that CCL21-ser is the essential player in maintaining T lymphocyte organization in the white pulp. However, we cannot rule out the possibility that MCMV inhibits additional, unidentified factors that regulate T cell localization in the spleen. Interestingly, human CMV has also been reported to modulate CCR7-dependent signaling. Human CMV infection of immature DC cells inhibits their ability to upregulate CCR7 expression, resulting in decreased chemotaxis in response to CCL19/CCL21 [33]. Therefore, both mouse and human CMV appear to specifically target this chemokine system, albeit by distinct mechanisms.

In addition to the dramatic disruption in lymphocyte organization observed after MCMV infection, significant alterations were also seen in MZ architecture. The MZM and MMM populations are one of the first cell types to encounter blood borne antigens/pathogens that enter the spleen via the MZ sinus, and are likely to play an important role in antigen capture and presentation. In addition, these MZ macrophages have been shown to be the major early producers of type I interferon (IFNαβ) after herpes simplex virus infection [34]. MCMV infection disrupts MZ organization within 2–3 d post-infection, and these changes could be due to several mechanisms. One possible explanation for the inability to detect ER-TR9^+^ MZM could be that ER-TR9 is downregulated from the cell surface and subsequently degraded during MCMV infection. ER-TR9 has recently been identified as the SIGN-R1 lectin (NP_573501) [35], a homologue of human DC-SIGN. DC-SIGN mediates infection of DC by human CMV through the binding of the viral glycoprotein B [36], and SIGN-R1 is internalized from the surface of MZM after binding to dextran or polysaccharides of Streptococcus pneumonia [37]. However, the loss of ER-TR9 expressing cells might be linked to the specific downregulation of CCL21-ser following MCMV infection, in agreement with the recent observation that *plt* mice have decreased numbers of MZM [31]. As potential support for this mechanism, we observed a ~9-fold reduction of ER-TR9 mRNA in the spleen 3 days following MCMV infection (data not shown), suggesting that this macrophage population may not be present in the spleen at this time point. The exact reason for the disruption of MOMA-1 + MMM in the MZ after MCMV infection is unknown at this point, but the broader, more disorganized pattern is not unlike that recently reported in mice lacking a receptor for sphingosine-1-phosphate-3 [38].

The dramatic effects on splenic architecture seen during viral infection raise the question of whether these events are specific to MCMV. Lymphoid tissues are dynamic structures that show alterations in both the hematopoietic and stromal compartments during immune responses. Infection of mice with other pathogens such as lymphocytic choriomeningitis virus [39] and Leishmania donovani [40] has been reported to alter lymphoid tissue architecture, but these changes occur at significantly later times after infection (weeks to months) and appear somewhat more “nonspecific” due to the loss/death of cell populations in the MZ or stroma. Kataki and colleagues demonstrated that the architecture of the lymph node changes after immunization with inert antigen with a strong adjuvant resulting in alteration of the boundary-separating T and B cells, and changes in the fibroblast reticular network [41]. Thus, for some tissue remodeling the presence of a replicating pathogen is not a prerequisite. Interestingly, changes in ER-TR7 expression were recapitulated to some extent in cultured lymph node fibroblastic reticular cell lines through activation of LTαβ and TNF signaling pathways [41]. Therefore, the changes in lymphoid tissue architecture during MCMV infection may represent viral exacerbation of “normal” host processes regulated by TNF-family cytokines. Nevertheless, it seems likely that MCMV may also employ specific strategies to disrupt lymphoid tissue architecture, which may facilitate replication, dissemination, and establishment of viral persistence. Support for this hypothesis comes from the fact that the disruption in architecture was most dramatic during the peak of acute viral replication, with recovery of lymphoid tissue architecture and CCL21 expression as MCMV replication was controlled.

MCMV-induced alterations in lymphoid tissue architecture were accompanied by specific enhancement of stromal cell expression of gp38. The restricted expression of gp38 to stromal cells located in the PALS of the white pulp has been known for some time [21]. Expression of this mucin in the PALS is defective in mice deficient in LTα [30]. The concurrent reduced levels of CCL21 and CCL19 expression in the spleens of LTα deficient mice is thought to be due to a defect in the maturation of PALS stromal cells, which co-express these chemokines and gp38 [2,30]. Gp38 analyzed by immunohistochemistry is undetectable in LTα-deficient mice [30], yet only a 2-fold loss of mRNA is observed, raising the possibility that stromal cells outside the T cell zone may express gp38 that is either not detectable by standard immunohistochemical techniques, or the cellular localization of gp38 may be regulated post-transcriptionally by LTαβ signaling (as was recently shown for the regulation of ER-TR7 expression [41]). The selective advantage of altering gp38 expression by either the host or the pathogen is unknown.

The LTαβ-LTβR system plays an essential role in the development and maturation of the splenic architecture, but may provide a limited contribution in the adult organ. Pharmacological inhibition of LTαβ signaling in adult mice only reduces CCL21 expression by 2-fold in the spleen [32]. Moreover, the homeostatic maintenance of splenic CCL21 expression in adult mice can be restored by bone marrow reconstitution with LTα-deficient bone marrow [30]. In contrast, pharmacological inhibition of LTαβ signaling in neonatal mice dramatically decreases splenic CCL21 expression (5-fold to 20-fold reduction) [30], and LTα or LTβ deficient mice are severely reduced (20-fold to 30-fold) in CCL21 expression. Taken together, these results support a model where LTαβ is critical during the embryonic development and postnatal maturation of CCL21 producing stromal cells, but once this cell population is “established” the contribution of LTαβ in maintaining CCL21 expression diminishes. Administration of an agonistic antibody to LTβR at the time of MCMV infection partially restored expression of CCL21-ser in the spleen and promoted increased localization of adoptively transferred naïve T cells into the PALS. These results indicate that although the LTαβ-CCL21 signaling “axis” may not maintain CCL21-ser expression in the adult spleen, the pathway can be modulated pharmacologically during a virus infection, as demonstrated previously in uninfected mice [12,13]. Importantly, these data also highlight targeting the LTβR as a potential avenue for manipulation of chemokine expression and potential restoration of some aspects of lymphoid tissue structure in the context of viral infection. This approach might prove useful in contexts where congenital or reactivated CMV infection poses significant clinical risks.

## Materials and Methods

### Mice and virus.

C57BL/6 (B6) mice were purchased from the Jackson Laboratories (Bar Harbor, Maine, United States). LTα-deficient mice were described previously [42] and were bred at LIAI. Female mice aged 6–8 wk were used. MCMV (Smith strain) and MCMV-GFP (Gift from J. Hamilton) [28] were prepared in salivary glands and infectious virions determined by plaque assay on NIH 3T3 cells as described in [43]. Virus was administered to mice via the intraperitoneal route.

### Confocal microscopy.

Spleens were removed, embedded in Tissue-Tek (Sakura Finetek USA, Torrance, California, United States), frozen on dry ice and stored at −80 °C. Cryostat sections (6–8-μm thick) were fixed in paraformaldehyde (4%) (for CCL21 and GFP staining) or ice-cold acetone for 10 min, rehydrated in PBS, and treated for 30 min with PBS 1% blocking reagent (PBS-BR) (Boehringer Mannheim, Mannheim, Germany) or PBS-BR containing 5% goat (for staining of CCL21) or rabbit serum (for GFP) to block nonspecific binding.

Cryosections were washed in PBS and stained for 60 min with the following uncoupled, biotinylated, or FITC-coupled primary antibody in PBS-BR containing 0.1% of saponin for anti-CCL21 (R&D Systems, Minneapolis, Minnesota, United States) and GFP Alexa-Fluor 647-conjugated rabbit anti-GFP (1:200) (Molecular Probes, Eugene, Oregon, United States) or in PBS-BR for RB6-8C5 (anti-Ly6G and –Ly6C), 30-H12 (anti-CD90.2), RA3-6B2 (anti-CD45R/B220), BP-3 (anti-BP-3/CD157), MECA-367 (anti-MAdCAM), and MEC 13.3 (PECAM-1/anti-CD31) (all from BD Pharmingen, La Jolla, California, United States); MOMA-1 (anti-MMM), ERTR-9 (anti-MZM), and ER-TR7 (BMA Biomedicals, Augst, Switzerland); 8.1.1 (anti-gp38; kind gift of A. G. Farr, University of Washington, Seattle, Washington, United States). Sections were washed in PBS and incubated for 30 min with the secondary detection reagents (in PBS-BR or PBS-BR containing 0.1% of saponin (CCL21)) streptavidin-Cy3 (1:200) (Zymed Laboratories, Invitrogen, Carlsbad, California, United States), streptavidin-APC (1:200), biotinylated anti-mouse IgG2b (1/50) to BP-3, biotinylated anti-mouse IgG2a (1/50) to MECA-367 and MEC 13.3 (BD Pharmingen), Alexa-Fluor 594-conjugated chicken anti-goat IgG (1:200) to CCL21 (Molecular Probes), and Cy-3-conjugated goat anti-syrian hamster IgG (1:200) to gp38 (Jackson Immunoresearch Labs). Sections were washed in PBS and incubated for 30 min with streptavidin-APC and streptavidin-Cy3 (1:200) tertiary detection reagents in PBS-BR 1% to detect BP-3 and ER-TR7 and MAdCAM and PECAM-1, respectively. The slides were washed in PBS and mounted in anti-fading GEL/MOUNT (Biomeda, Foster City, California, United States). In sections stained with two biotinylated antibodies, the excess biotin from the first antibody was blocked with excess avidin (Vector Laboratories, Burlingame, California, United States) before incubation of the sections with a second biotinylated antibody. Digitized images were acquired using a confocal imaging station (Bio Rad MRC-1024 confocal microscope [Bio-Rad Laboratories, Hercules, California, United States] with a Krypton/Argon Ion Laser and Nikon TE 300 inverted microscope [Nikon, Tokyo, Japan]) and processed with Photoshop Software (Adobe Systems, San Jose, California, United States).

### Quantitative PCR.

Levels of gene expression were measured by quantitative RT-PCR using a Stratagene Mx 4000 and SYBR green reagent (BioRad). Spleens from mock or MCMV-infected mice were snap frozen in liquid nitrogen before homogenization in Trizol reagent (Invitrogen) and total cellular RNA isolated and quantified before performing DNAse digestion and reverse transcription as described [19]. Primers to specifically detect CCL21ser and CCL21leu were described previously [7]; all other primer sequences are available upon request.

### Splenic stromal cell fractionation.

Spleens from mock or MCMV-infected mice were removed aseptically at day 3 post-infection. Spleens were perfused with 1ml/spleen of collagenase (100 U/ml) (CLSIII, Worthington Biochemical, Freehold, New Jersey, United States), resuspended in Hanks' Balanced Salt Solution (HBSS, 100U/ml) (Invitrogen) containing Ca^2+^ and Mg^2+^. After perfusion with collagenase, spleens were incubated for 30 min at 37 °C in collagenase (400 U/ml) solution (1 ml/spleen). Spleens were then extruded through a 70-μm nylon filter strainer (BD Falcon, BD Biosciences, Franklin Lakes, New Jersey, United States). After extrusion, the cell strainer was washed with sterile PBS (Invitrogen) followed by the direct lysis of filter-bound “stromal cells” with 1 ml of Trizol (Invitrogen). Cells that were extruded through the filter (“hematopoietic cells”) were washed with sterile PBS (Invitrogen,) and then lysed in Trizol (Invitrogen) (10^7^ cell/ml). Total cell RNA isolation and subsequent reverse transcription was performed according to the manufacturers' protocols and as described in [19]. The relative “contamination” of filter-bound stromal cells with hematopoietic cells was estimated by analysis of genes preferentially expressed in the hematopoietic cells, including CD80, IL-7Rα, LTα and LTβ. Using these markers, mRNA expression in the hematopoietic fraction was found to be 4.4×, 5.3×, 6.3× and 3.5× enriched in each marker, respectively, relative to the filter retained stromal cell fraction. IL-7 and gp38 mRNAs, two genes expressed preferentially in the splenic stroma, were present at 14× and 17× higher levels in the stromal cells, respectively. These data indicated the filter-bound stromal fraction from uninfected mice contains ~15%–30% contaminating hematopoietic cells, and 4%–7% contaminating stromal cells in the hematopoietic fraction based on the assumption that these marker genes are expressed exclusively in their respective compartments.

### Flow cytometry.

Cells were analyzed by flow cytometry with a FACSCalibur (BD Biosciences). The cells were preincubated with saturating amount of 2.4G2 (a rat anti-mouse FcR mAb; BD Pharmingen) for 10 min before staining to prevent Ab binding to FcR and further labeled with PE-coupled 1A8 (anti-Ly-6G) and 145–2C11 (anti-CD3ɛ or PerCP-coupled M1/70 (anti-CD11b) and RA3-6B2 (anti-CD45R/B220), or APC-coupled DX5 (anti-CD49b/Pan NK cells) and HL3 (anti-CD11c) Abs from BD Pharmingen [44]. Cells were gated according to size and scatter to eliminate dead cells and debris from analysis.

### T cell transfer.

Splenic T cells were purified by negative selection using a “Pan T Cell Kit” (Miltenyi Biotech, Auburn, California, United States) following the manufacturer's protocol, and were labeled for 10 min at 37 °C with 1 μM CFSE (5- [and 6-] carboxyfluorescein diacetate succinimidyl ester; Molecular Probes). CFSE-labeled T cells (10^7^) were injected via the lateral tail vein, and spleens were recovered 2 h later for immunohistochemistry and flow cytometric analysis. MCMV-infected mice receiving anti-LTβR agonistic antibody (3C8) were injected once with 100 μg i.p. at the time of infection.

Spleen sections were stained with MOMA-1 and Thy1.2 to delineate the white pulp boundary and confirm the presence of T cells preceding the analysis of CFSE-labeled cell number within the white pulp. Immunofluorescent images were recorded by a digital fluorescence microscopy system (Intelligent Imaging Innovations, Denver, Colorado, United States), including a Zeiss Axiocam Mrm charge-coupled device camera and a Zeiss Axiovert 200M microscope (Carl Zeiss Microimaging, Thornwood, New York, United States) and emission filter wheels (Sutter Instrument, Novato, California, United States) with narrow band optical filters (Chroma Technology, Rockingham, Vermont, United States). All components were controlled by SlideBook software (Intelligent Imaging Innovations), and the same software was used for image capture and analysis of the number of CFSE(+) T cells within the white pulp areas.

## Supporting Information

Figure S1 Analysis of Splenic Hematopoietic Cells in MCMV Infected Mice For all analyses, mice were infected with 3.2 × 10^5^ pfu MCMV-Smith or MCMV-GFP.
(A) Spleens from mock and MCMV-infected mice were analyzed by flow cytometry 48 h and 72 h post-infection. The changes in the granulocyte (gran,Gr-1^hi^ F4/80^−^), macrophage (macro, Gr-1^lo^ F4/80^hi^ CD11c^lo^) and DC (DC, CD11c^hi^ F4/80^lo^) populations were analyzed. Infection with either MCMV-Smith or MCMV-GFP induced the recruitment of granulocytes, the loss and/or alteration in the cell surface phenotype of macrophages (F4/80 downregulation and CD11b upregulation, see also (B)), and loss/alteration of DC. Also note the increase of autofluorescent cells (moving along the diagonal of the dot plot) in MCMV-infected mice.
(B) The expression of CD11b on macrophage (Gr-1^lo^ F4/80^hi^ CD11c^lo^) and “MCMV-macrophage” (Gr-1^lo^ F4/80^int^ CD11c^lo^) populations was analyzed in mock and MCMV-infected mice at 48 h post-infection (similar results were also seen at 72 h, unpublished data). The dot plots show that the expression of F4/80 decreased on the cell population presumed to be splenic macrophages in MCMV-infected mice (i.e., “MCMV-macrophages”) as reported previously [29], while the expression of GR-1 in these cells remained the same. However, the altered expression of CD11b in macrophages from these three experimental groups also raised the possibility that this is a distinct cell population only seen in the spleen of MCMV-infected mice, potentially recruited from the blood or bone marrow. Mock (red), MCMV-Smith (blue), and MCMV-GFP (green). (C) The percentage of GFP-expressing cells was determined in individual cell populations from spleens of MCMV-GFP–expressing mice (a summary of data is presented in Table 1). For this analysis, GFP-expressing cells were defined as cells that displayed higher levels of fluorescence in the FL-1 channel when compared with mice infected with MCMV-Smith virus (not mock-infected mice). The increase in the autofluoresence occurred in DC, granulocyte, and NK cell populations following infection with MCMV-Smith. DC and granulocytes were phenotypically defined as in (A). For analysis of macrophages, FL-1 fluorescence displayed in histograms was analyzed by gating on the macrophages (Gr-1^lo^ F4/80^hi^ CD11c^lo^) in mock-infected mice and compared with the “MCMV-macrophages” (Gr-1^lo^ F4/80^int^ CD11c^lo^). GFP expression in NK cells (DX5^+^CD3^−^), and lymphocytes (72 h only) was also analyzed. The higher percentage of cells expressing GFP as fraction of the total splenocytes observed at 72 h after infection (9.9%) compared with 48h (1.6%) is considered in the context of the increased lymphocyte death at this time point (shown in Figure 4B). The high level of FL-1 autofluorescence in the DC from mice infected with MCMV-Smith at both 48 h and 72 h post-infection may be due to activation and/or apoptosis of these cells, and a change in the “tightness” of this gated population is evident in MCMV-infected mice. Virtually all splenic DC matured (defined by upregulation of CD80, CD86, CD40, and MHC II) upon infection with MCMV at this dose by 16 h post-infection (unpublished data). Granulocytes expressed higher levels of GFP at 72 h compared with 48 h after infection, suggesting that MCMV-GFP replicates in these cells. However, increased phagocytosis of GFP protein or GFP-expressing cells by granulocytes cannot be excluded. Data shown is from splenocytes pooled from two individually infected mice, and is representative of the results seen in three independent experiments.
(1.0 MB PPT)

### Accession Numbers

The accession numbers for the following are from the National Center for Biotechnology (http://www.ncbi.nlm.nih.gov/entrez/query.fcgi): CCR7 (NP_031745); ELC/MIP-3β (AAH25130); CCL21-ser(a) (P84443); CCL21-leu(b) (P84444); CXCR5 (AAH64059); CXCL13 (O55038); TNFSF1 (P_034865); TNFSF3 (NP_032544); TNFSF2 (P06804); TNFRSF3 (NP_034866); TNFRSF3 (NP_034866); T1α/podoplanin (CAC16152); PECAM-1 (Q08481); and MAdCAM (NP_038619).

## Abbreviations

CMV: cytomegalovirus
DC: dendritic cells
EC: endothelial cells
LT: lymphotoxin
MAdCAM: mucosal addressin cell adhesion molecule
MCMV: mouse cytomegalovirus
MMM: marginal metallophilic macrophages
MZM: marginal zone macrophages
NK: natural killer
PALS: periarteriolar lymphoid sheath
PECAM: platelet–endothelial-cell adhesion molecule
*plt*: paucity-of-lymph-node T cells
